# Supplementary figures and images for: TGF-β expressed by M2 macrophages promotes wound healing by inhibiting TSG-6 expression by mesenchymal stem cells
Source: PLoS One. 2025 Apr 21;20(4):e0316692. doi: 10.1371/journal.pone.0316692 (PMC12011265; doi:10.1371/journal.pone.0316692)

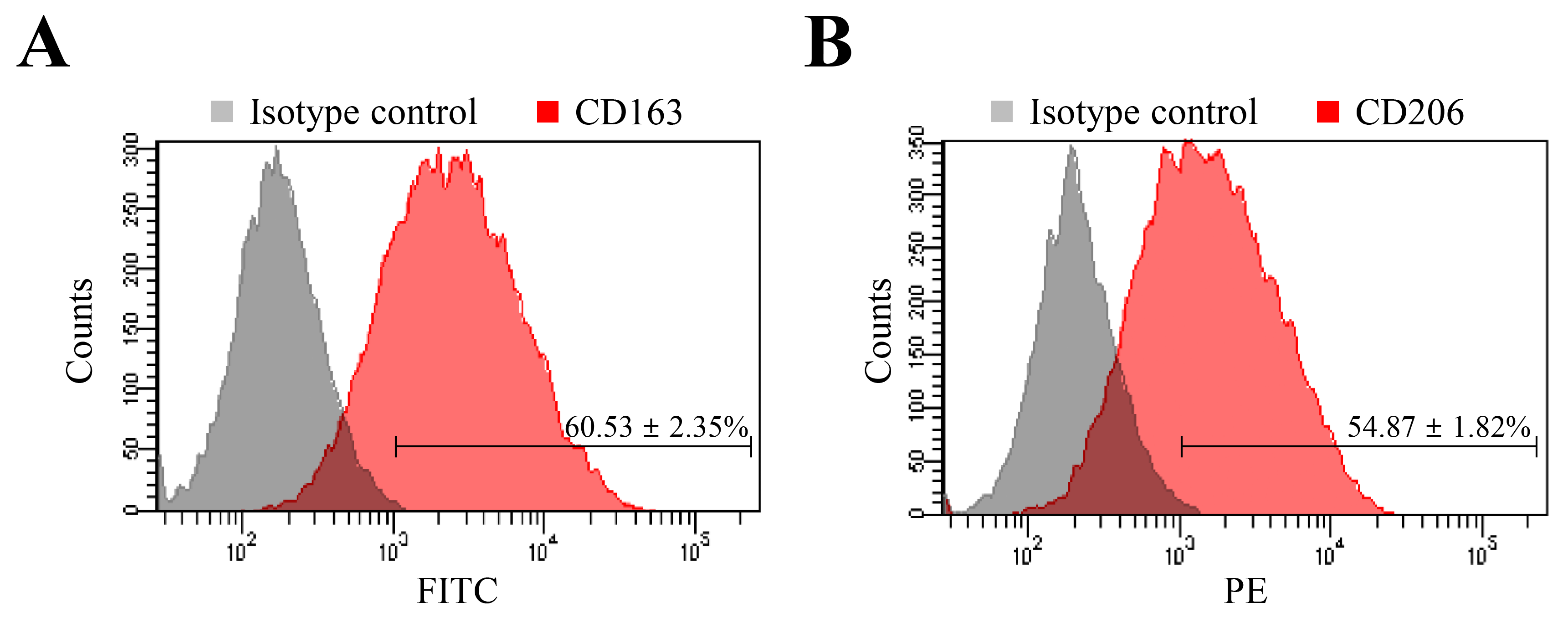

Supplement: S1 Fig — To differentiate M2 macrophages, PMA-treated THP-1 cells were treated with IL-4 (20 ng/mL) and IL-13 (20 ng/mL) for 48 h. Cells were then harvested using trypsin treatment and centrifugation. Macrophages were stained with fluorescein isothiocyanate (FITC)-conjugated anti-human CD163 antibodies or phycoerythrin (PE)-conjugated anti-human CD206 antibodies (both from BD Biosciences, San Jose, CA, USA) in the dark for 20 min at room temperature. FITC or PE-conjugated mouse immunoglobulin G was used as the isotype control at the same concentration. The fluorescence intensity of the cells was evaluated using flow cytometry (FACS Aria III Cell Sorter (BD Biosciences, Franklin Lakes, NJ, USA), and the data were analyzed using the FACSDiva Software v8.5 (BD Biosciences). (TIF) [file pone.0316692.s001.tif]

Fig 1B

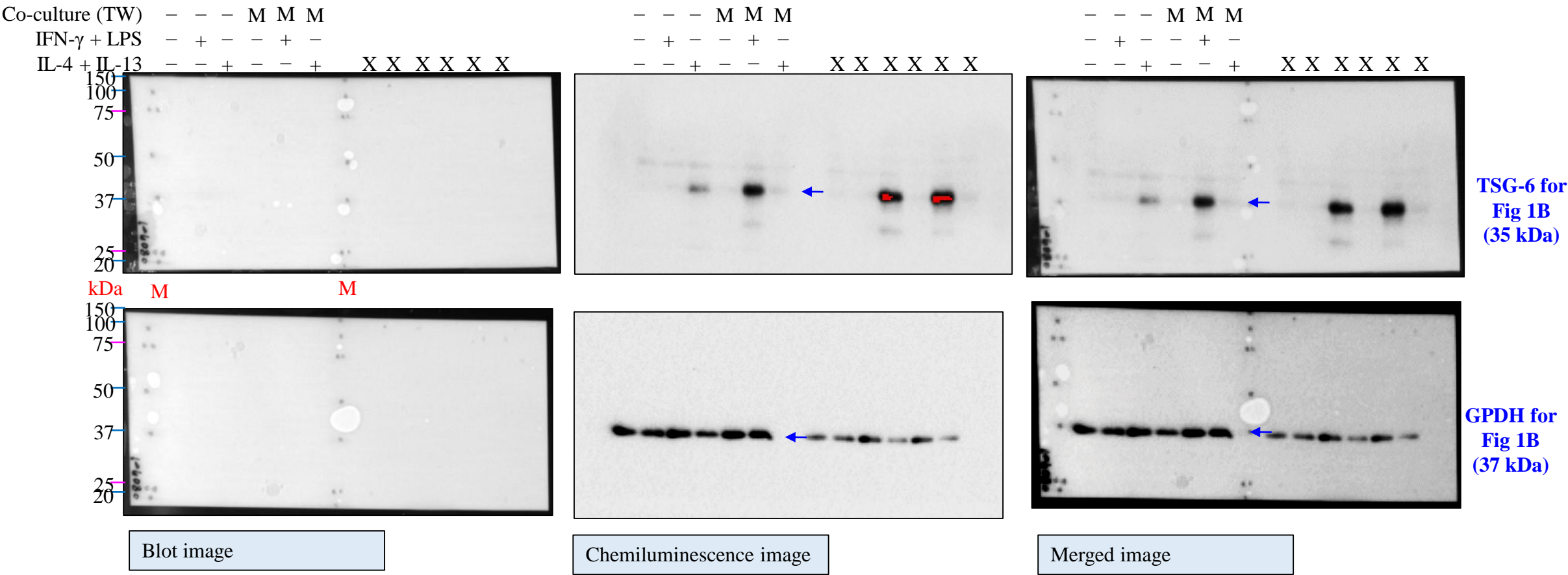

Fig 2C

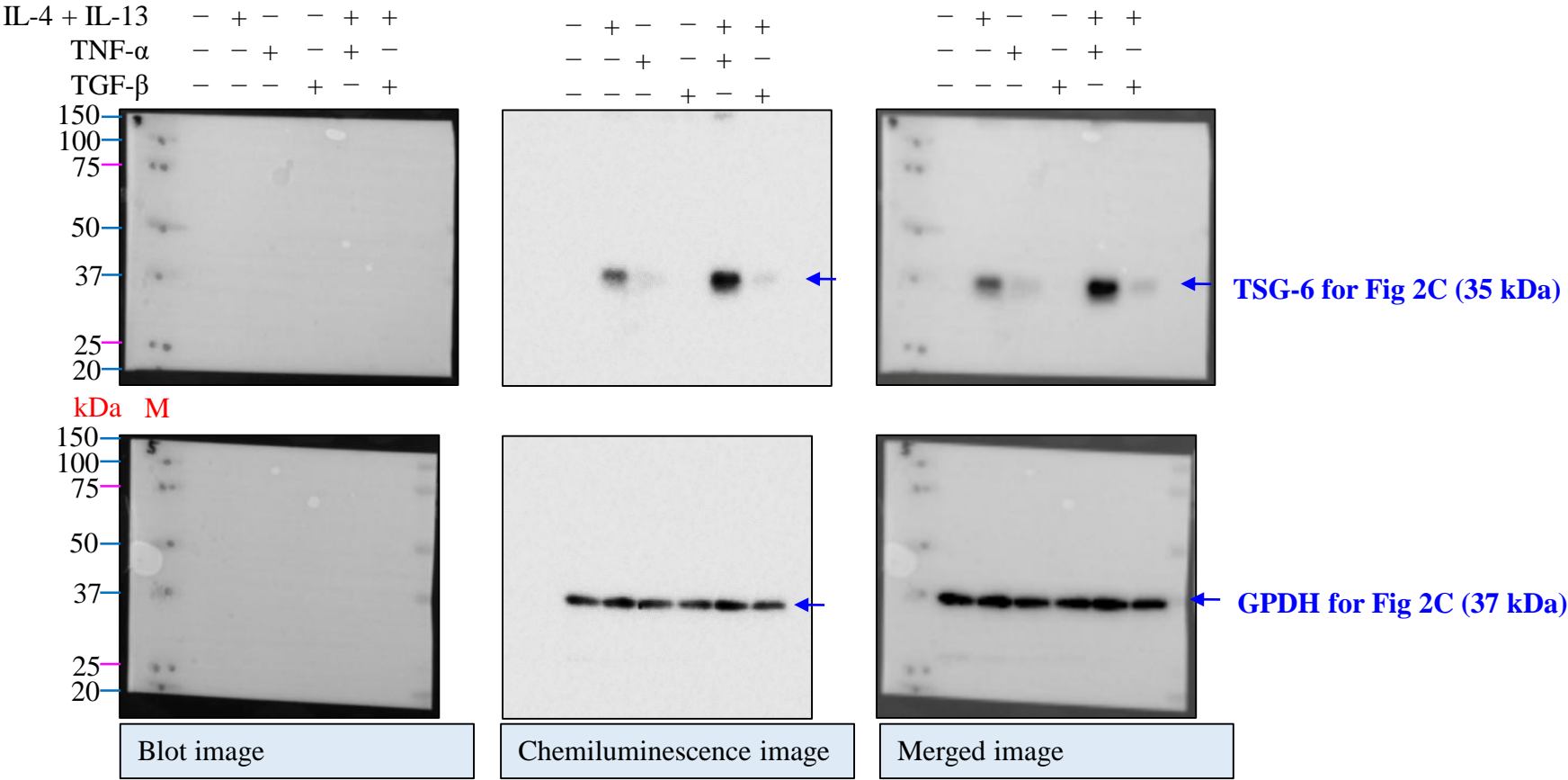

Fig 2D

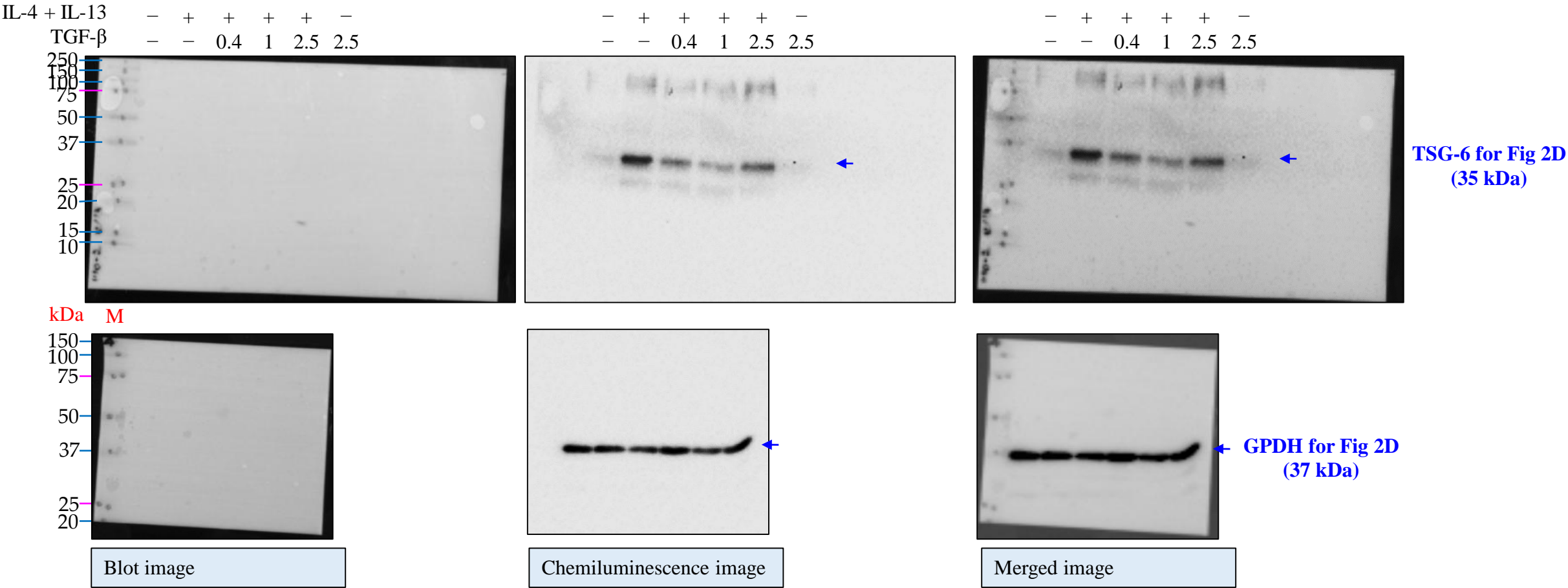

Fig 3A; 15 m

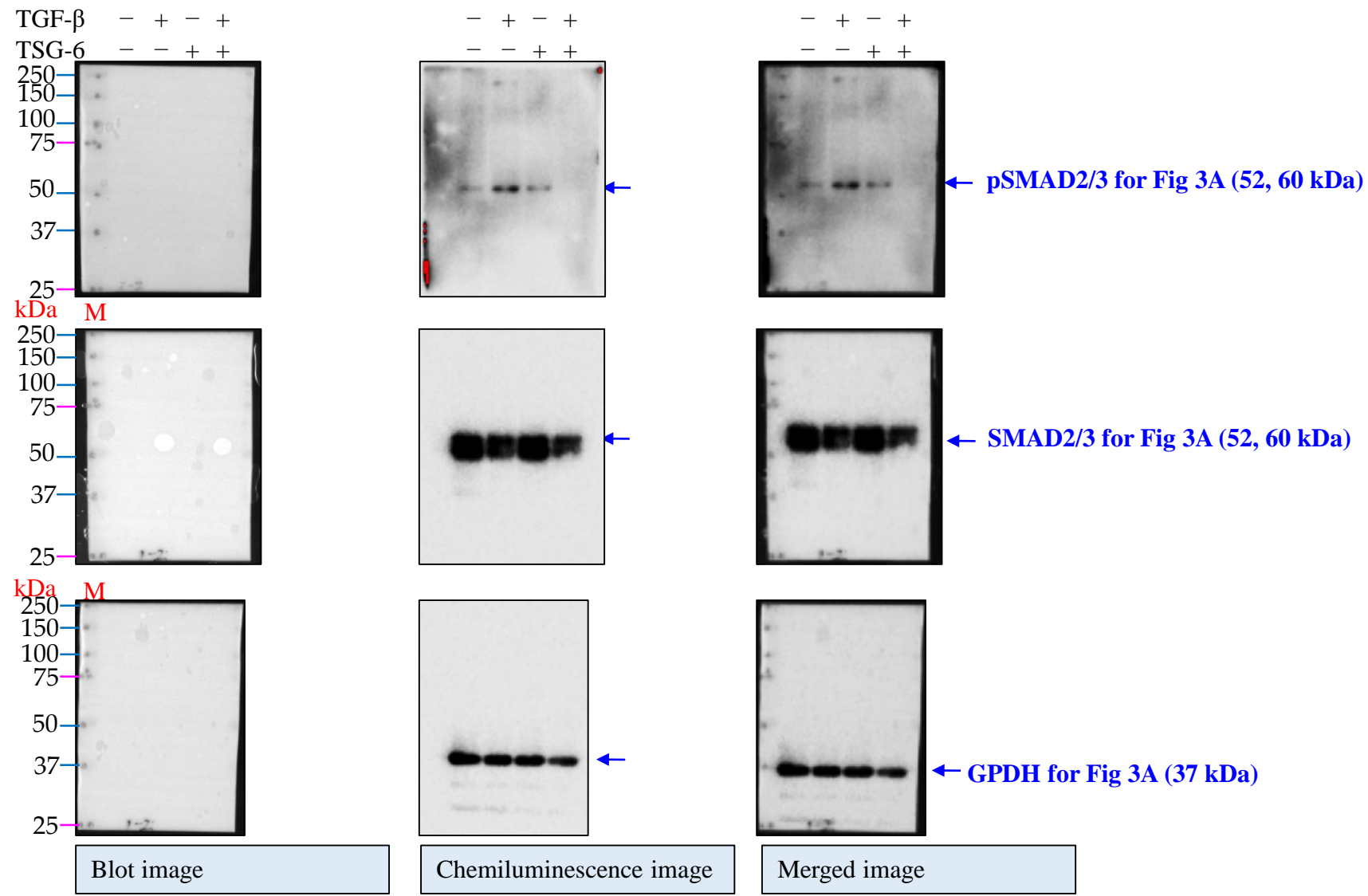

|              |   |   |   |   |
|--------------|---|---|---|---|
| TGF- $\beta$ | - | + | - | + |
| TSG-6        | - | - | + | + |

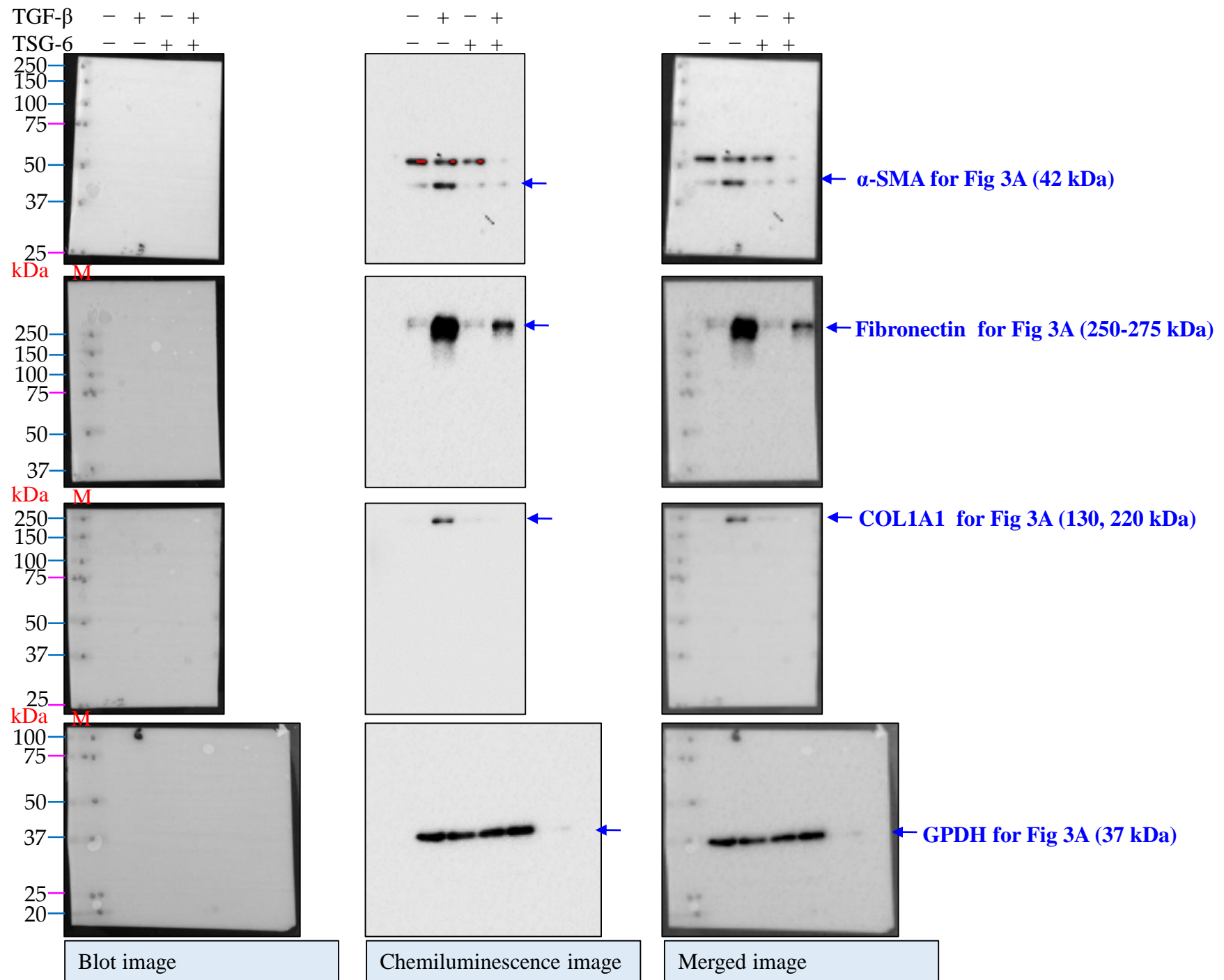

Supplement: S1 Raw images — (PDF) [file pone.0316692.s002.pdf]
